# Gene expression of *Lactobacillus plantarum* and the commensal microbiota in the ileum of healthy and early SIV-infected rhesus macaques

**DOI:** 10.1038/srep24723

**Published:** 2016-04-22

**Authors:** Benjamin L. Golomb, Lauren A. Hirao, Satya Dandekar, Maria L. Marco

**Affiliations:** 1 Department of Food Science and Technology, University of California, Davis, CA, USA; 2 Department of Medical Microbiology and Immunology, University of California, Davis, CA, USA

## Abstract

Chronic HIV infection results in impairment of gut-associated lymphoid tissue leading to systemic immune activation. We previously showed that in early SIV-infected rhesus macaques intestinal dysfunction is initiated with the induction of the IL-1β pathway in the small intestine and reversed by treatment with an exogenous *Lactobacillus plantarum* strain. Here, we provide evidence that the transcriptomes of *L. plantarum* and ileal microbiota are not altered shortly after SIV infection. *L. plantarum* adapts to the small intestine by expressing genes required for tolerating oxidative stress, modifying cell surface composition, and consumption of host glycans. The ileal microbiota of *L. plantarum-*containing healthy and SIV+ rhesus macaques also transcribed genes for host glycan metabolism as well as for cobalamin biosynthesis. Expression of these pathways by bacteria were proposed but not previously demonstrated in the mammalian small intestine.

Adaptations of bacteria to the small intestine are currently largely uncharacterized for either healthy or diseased individuals. The lack of information on the microbiota at this site in the digestive tract is remarkable considering its significance for nutrient uptake and immune function^1^,^2^. Nutrient absorption occurs in the small intestine and the resident microbiota contribute to both nutrient availability and metabolism of diet (e.g. sugars and amino acids) and host (e.g. bile) derived compounds^3^,^4^,^5^. Intestinal bacteria might also synthesize vitamins, amino acids, and other cofactors that are absorbed through small intestine enterocytes^3^,^6^. Finally, the small intestine microbiota are critical for priming of the naïve immune system in the gut-associated lymphoid tissue (GALT)^7^.

Of equal relevance to understanding the indigenous small intestine microbiota is knowledge on the behaviours of dietary bacteria during passage through the mammalian gut. Dietary *Lactobacillus* species, in particular, are regarded to be more active in the upper digestive tract as opposed to the large intestine^7^. *Lactobacillus plantarum* is a species that can be consumed in high numbers in fermented foods and probiotic supplements. *L. plantarum* strain WCFS1 was shown to alter the human small intestine by reinforcing intestinal epithelial tight junctions^8^ and skewing immune responses towards a regulatory phenotype^9^. These effects are not limited to this strain as other lactobacilli are known drive host transcriptional responses in the small intestine towards a more tolerant phenotype^10^.

In efforts to identify the cell products of *L. plantarum* WCFS1 that are responsible for modifying epithelial and immune cells in the digestive tract, it was found that strains of this species exhibit similar transcriptome programming in the large intestines of healthy humans and mice^11^. Predominant adaptations of *L. plantarum* WCFS1 to the distal intestine are the induction of carbohydrate metabolism and cell surface modulating pathways^12^,^13^,^14^. However, the response of *L. plantarum* is dynamic and influenced by the intestinal compartment and the diet and inflammatory status of the host^13^,^15^. *L. plantarum* WCFS1 was significantly better at preventing colitis when consumed in a diet enriched in sucrose and animal fat as opposed to a low-fat, high plant-polysaccharide containing chow^15^. Intriguingly, *L. plantarum* stimulation of higher IL-10 to IL-12 ratios in mouse colonic tissues was independent of host diet^15^ suggesting that certain *in vivo* expressed properties of *L. plantarum* are highly conserved and function independently of diet and microbiota-associated factors.

Chronic human immunodeficiency virus (HIV) infection results in changes to the GALT including a loss of gut-resident CD4^+^ T cells, epithelial barrier disruption and bacterial translocation, and ultimately systemic immune activation^16^,^17^,^55^. Under normal conditions, the intestinal microbiota provides the host with nutrients, protects against enteric pathogens, and aids in maintaining immune homeostasis. However, the microbiota in chronically infected HIV patients is perturbed^18^,^19^ suggesting that it may respond to changing conditions in the GALT. Less understood are the early effects of HIV infection on the gut epithelium prior to CD4^+^ T cell depletion. We previously showed that shortly (2.5 d) after infecting rhesus macaques with simian immunodeficiency virus (SIV), the IL-1β pathway is upregulated in Paneth cells resulting in damage to the epithelial barrier^20^. Injection of *L. plantarum* WCFS1 into the ileal lumen reduced IL-1β levels likely through inhibition of the NF-κB pathway leading to restored barrier function^20^. However, effects of *L. plantarum* in the ileum were independent of infection status, and therefore it remains unknown whether the *L. plantarum* inoculant was exposed to different conditions in the lumen of SIV+ macaques requiring compensatory responses for its survival and function.

Herein, we investigated whether *L. plantarum* gene expression in the small intestine was altered during the early stages of SIV infection. This was accomplished by injecting and incubating *L. plantarum* for 5 h directly in the ileal lumen of our previously established ligated loop model which allows a direct view into this difficult to reach site in the gastrointestinal tract^20^. Using transcriptome profiling, we compared gene transcript levels of *L. plantarum* WCFS1 in the ileum of rhesus macaques 2.5 d after SIV infection to transcripts produced by this strain in healthy animals. Comparisons were also made to the transcriptomes of *L. plantarum* reference cultures. Concurrently, we addressed whether the commensal microbiota of the *L. plantarum*-treated macaques was affected by the presence of the virus. Our results indicate that at this very early stage of viral infection, neither *L. plantarum* nor the commensal microbiota respond at the transcriptional level to SIV presence or changes in the ileal environment. Microbial gene expression patterns in the rhesus macaque small intestine were not previously observed in other mammalian hosts and some of those behaviours were shared with *L. plantarum*.

## Results

### *L. plantarum* gene expression is not altered by early SIV infection

**Figure 1:**
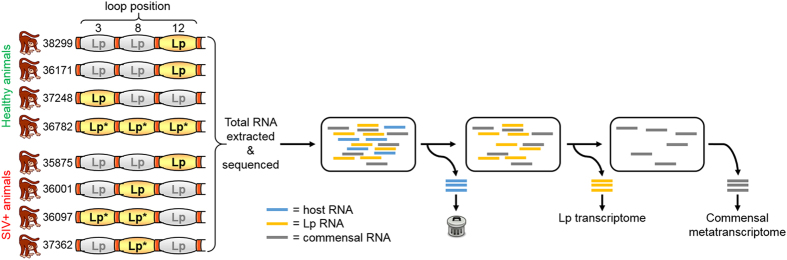
*L. plantarum* transcriptome and commensal metatranscriptome sampling scheme. Total RNA was extracted from the luminal contents of ileal loops injected with *L. plantarum* from 4 healthy animals and 4 SIV+ animals, each with 3 loops, for a total of 24 loops. Loops from which RNA of sufficient quality was obtained and sequenced are highlighted in yellow. Loops from which metatranscriptomes were analyzed are denoted with an asterisk (*). Following sequencing, reads that aligned to the rhesus macaque genome were discarded. The remaining reads were then aligned to the *L. plantarum* genome and reads originating from the commensal microbiota were assembled into contigs for metatranscriptome analysis.

**Figure 2:**
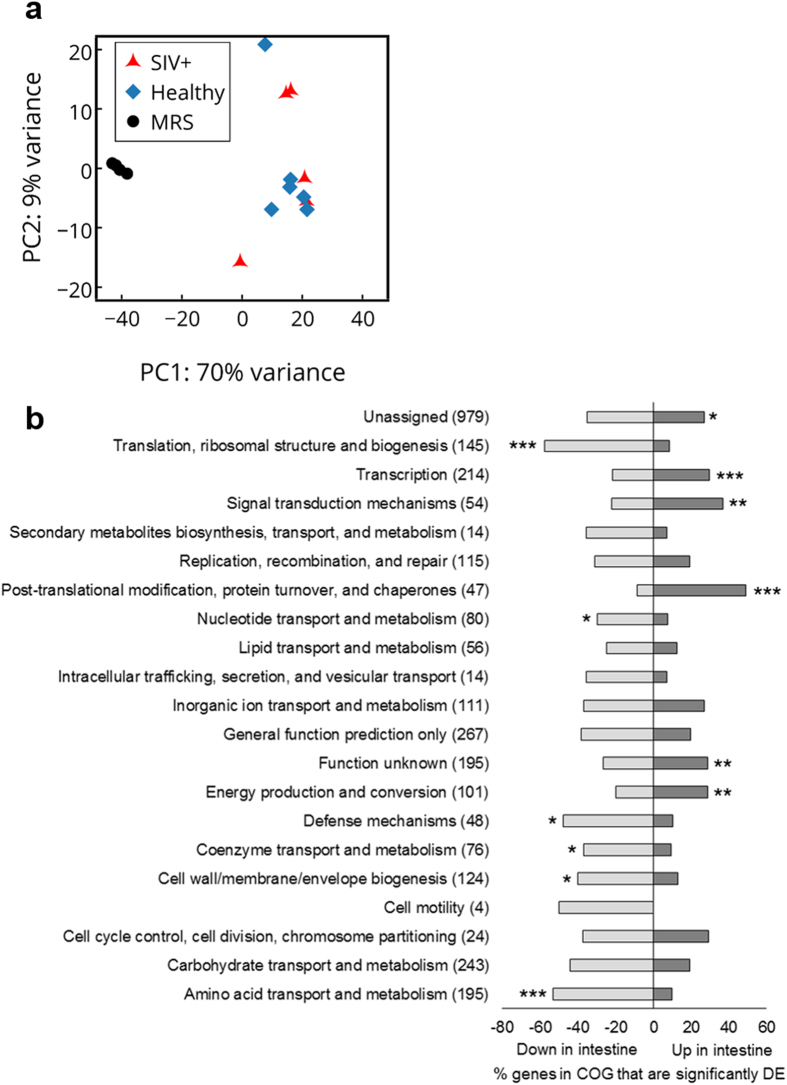
Principle component analysis (PCA) of *L. plantarum* transcriptome profiles and COG categories. (**a**) The first and second components are shown for each plot and the % on each axis explains the variance by each component. No clustering was observed when comparing *L. plantarum* transcriptomes from SIV+ to healthy samples however distinct clustering was observed when comparing *L. plantarum* transcriptomes from healthy and SIV+ intestinal samples to *L. plantarum* transcriptomes from MRS samples. (**b**) The percentage of *L. plantarum* genes in COG categories that were differentially expressed in the intestine of healthy rhesus macaques as compared to MRS is shown. The total number of genes in each COG is indicated in parentheses. Genes are considered differentially expressed (DE) if there was at least a 2-fold change in expression and an FDR-adjusted *P* < 0.05. COG categories that are significantly overrepresented according the χ^2^ test compared to the total number of genes in the genome are indicated by an asterisk (*P < 0.05; **P < 0.005; ***P < 0.0005).

*L. plantarum* WCFS1 injected into ileal loops of SIV infected rhesus macaques caused a decrease in expression of IL-1β and increase in the repair of the intestinal epithelium^20^. To investigate whether *L. plantarum* responds differentially in the ileum in a manner that is dependent on SIV infection, we performed whole-genome transcriptome profiling for *L. plantarum* from the ileum of healthy and infected animals according to the scheme in Fig. 1. Principle component analysis (PCA) of the transcriptomes showed that *L. plantarum* gene expression was very similar in the healthy and early SIV-infected macaques (Fig. 2A). Remarkably, none of the 3106 *L. plantarum* genes measured were differentially expressed. This result showed that the transcriptomes of *L. plantarum* WCFS1 in the small intestine were highly reproducible. Secondly, the lack of change in *L. plantarum* gene expression confirmed that early stages of SIV infection do not modify *L. plantarum* behaviour in the intestine.

### *L. plantarum* adaptations for growth in the small intestine

Because of the remarkably consistent transcriptomes of *L. plantarum* WCFS1 in the ileal lumen of healthy and early SIV infected macaques, we next investigated how *L. plantarum* responds to the small intestine. To identify those changes the *in vivo* transcriptomes and those identified for exponential-phase *L. plantarum* WCFS1 cells in MRS laboratory culture medium were compared. This *in vitro* reference condition was selected because it facilitates comparisons to actively dividing cells and the results can be related to prior studies performed using this strain. Relative to exponential-phase *L. plantarum* WCFS1, the ileal transcriptome of this strain encompassed 687 *L. plantarum* genes (22.1% of the coding sequences) that were significantly induced and 1112 genes (35.8% of the coding sequences) significantly repressed in the ileum in healthy rhesus macaques (Supplementary Table 1). The same result was reached for comparisons between MRS-grown *L. plantarum* and early SIV+ macaques. This finding was expected based on direct comparisons of the *L. plantarum* ileal transcripts (Fig. 2A). Because the immunomodulatory effects of *L. plantarum* were not impaired by early SIV infection^20^ and *L. plantarum* did not modify its transcriptome depending on infection status, we next focused our attention on *L. plantarum* gene expression in healthy animals.

*L. plantarum* Clusters of Orthologous Groups (COGs) with significantly increased numbers of ileal induced genes in healthy animals included post-translational modification, protein turnover, and chaperones (23 genes, 49% of the total genes in the COG), signal transduction mechanisms (22 genes, 41% of the total genes in the COG), and transcription (81 genes, 41% of the total genes in the COG) (Fig. 2B). COGs significantly enriched with repressed genes included translation, ribosomal structure and biogenesis (99 genes, 68% of the total genes in the COG), amino acid transport and metabolism (120 genes, 62% of the total genes in the COG), and defense mechanisms (26 genes, 54% of the total genes in the COG) (Fig. 2B). These results were nearly identical for *L. plantarum* in the SIV+ rhesus macaques (Supplementary Fig. 1). Ileal responses of *L. plantarum* for carbohydrate metabolism, stress response, and extracellular composition are discussed in the following sections.

### Carbohydrate metabolism

*L. plantarum* is an exclusively saccharolytic organism that relies on the availability of mono-/di-saccharides and short-chain oligosaccharides for both energy metabolism and growth. Strikingly, very few *L. plantarum* WCFS1 carbohydrate metabolism genes were induced in the rhesus macaque ileum and those that were up-regulated can be employed to consume host glycans. Among the *in vivo* induced genes was *nanA* coding for an *N*-acetylneuraminate lyase required for sialic acid metabolism (induced 9.1-fold). Sialic acid [*N*-acetylneuraminate, abbreviated as Neu5Ac] is a nine-carbon compound and component of mucin that is utilized by gastrointestinal bacteria as an energy source. Certain phosphotransferase system (PTS) genes were also induced in *L. plantarum* including those annotated for mannose (*pts9* and *pts10*, 4.5- and 2-fold respectively) and *N*-acetylglucosamine/galactosamine (*pts19*, 8.8-fold) transport (Supplementary Table 1).

Among the nearly 50% of down-regulated genes in the carbohydrate metabolism COG in healthy (Fig. 2B) and SIV+ animals (Supplementary Fig. 1) were genes coding for enzymes required for the metabolism of carbohydrates likely encountered in the diet such as sucrose (*scrB*), trehalose (*treP*), galactosides (*lacA* and *melA*), and sorbitol (*srlD1*) (Supplementary Table 1). Similarly reduced were transcripts for PTS genes coding for the uptake of sugars such as those for β-glucosides (*pts15, pts30*, and *pts33*, 2 to 6.3-fold), oligosucrose (*pts1*, 6.3-fold), fructose (*pts31*, 3-fold), and galactitol (*pts36*, 4-fold) (Supplementary Table 1).

### Oxidative stress response in the ileum

*L. plantarum* expressed genes for responding to oxidative stress in the rhesus macaque ileum. Among the induced genes in the post-translational modification, protein turnover, and chaperone mechanism COG (Fig. 2B) were those coding for the class III stress regulon controlled by CtsR^21^,^22^. Many of the genes under control of CtsR were up-regulated including *clpP* (7.2-fold), *clpE* (12.4-fold), *clpB* (12.9-fold), *clpX* (3.6-fold), *clpL* (31.6-fold), and *hsp1* encoding a small heat shock protein (91.1-fold) (Supplementary Table 1). Similarly, transcripts for chaperones *groES* (2.9-fold) and *groEL* (2.4-fold) were increased and genes for nucleotide-binding proteins of the UspA (universal stress protein) family were the two most highly induced genes in the ileum [lp_1747 (277.5-fold) and lp_1701 (149.9-fold)] (Supplementary Table 1). Six other homologs of UspA (lp_1163, lp_1322, lp_2340, lp_2652, lp_2745, and lp_2877) were also highly overexpressed at 148.2-fold (Supplementary Table 1). Lastly, genes coding for thioredoxins (*trxA1-3* and *trxH*) were induced as well as four out of five genes for RNA polymerase (RNAP)-binding regulatory proteins (*spx1-5*) (Supplementary Table 1).

### Cell-surface associated proteins

Numerous L. plantarum genes responsible for products targeted to the cell surface were differentially expressed in the ileum. Extracellular and secreted proteins of the gut microbiota are important mediators of host-microbe interactions^23^. Among the *in vivo* induced genes was lp_1697 (14.6-fold) coding for an adherence protein with a chitin binding domain^24^. Conversely, genes for four cell-surface sortase-dependent proteins [mucus-binding proteins (lp_1643 and lp_3114), cell surface protein precursor (lp_2925), and mannose-specific adhesion (*msa*)]^25^, extracellular polysaccharide production [glycosylhydrolase (lp_1187), rhamnose biosynthesis (*rfbBCD*)], and four capsular polysaccharide gene clusters [*cps1A-J, cps2, cps3A-I,* and *cps4A-J*] were all significantly down-regulated in the ileum (Supplementary Table 1).

### The *L. plantarum*-treated rhesus macaque ileal metatranscriptome consists primarily of *Streptococcaceae* and *Pasteurellaceae*

**Figure 3:**
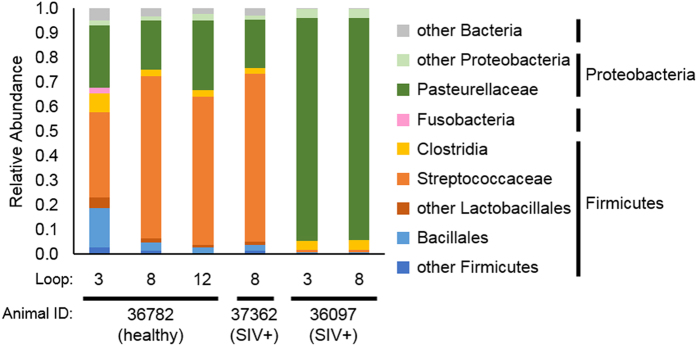
Relative abundances of bacterial families identified in ileal loop metatranscriptomes. Relative abundances were determined using MG-RAST using the best hit classification method against the M5nr database. A maximum e-value of 1e-5, a minimum identify of 60%, and a minimum alignment length of 15 amino acids for translated sequences was used.

Transcripts that were not mapped to *L. plantarum* or the rhesus macaque genomes were used for metatranscriptome assemblies (Fig. 1). Metatranscriptomes of sufficient reads (see Methods) were assembled for three different rhesus macaques administered *L. plantarum* (Fig. 1). These animals (1 healthy and 2 SIV+) were dominated by members of the *Streptococcaceae* and *Pasteurellaceae* families (Fig. 3). *Streptococcaceae* constituted the majority of the bacteria in the healthy animal (34 to 66% of the total) and one with SIV (68% of the total) whereas the other with SIV was overwhelmingly populated with bacteria from *Pasteurellaceae* family (90% of the total) (Fig. 3).

### Early SIV infection does not alter the rhesus macaque ileal metatranscriptome

**Figure 4:**
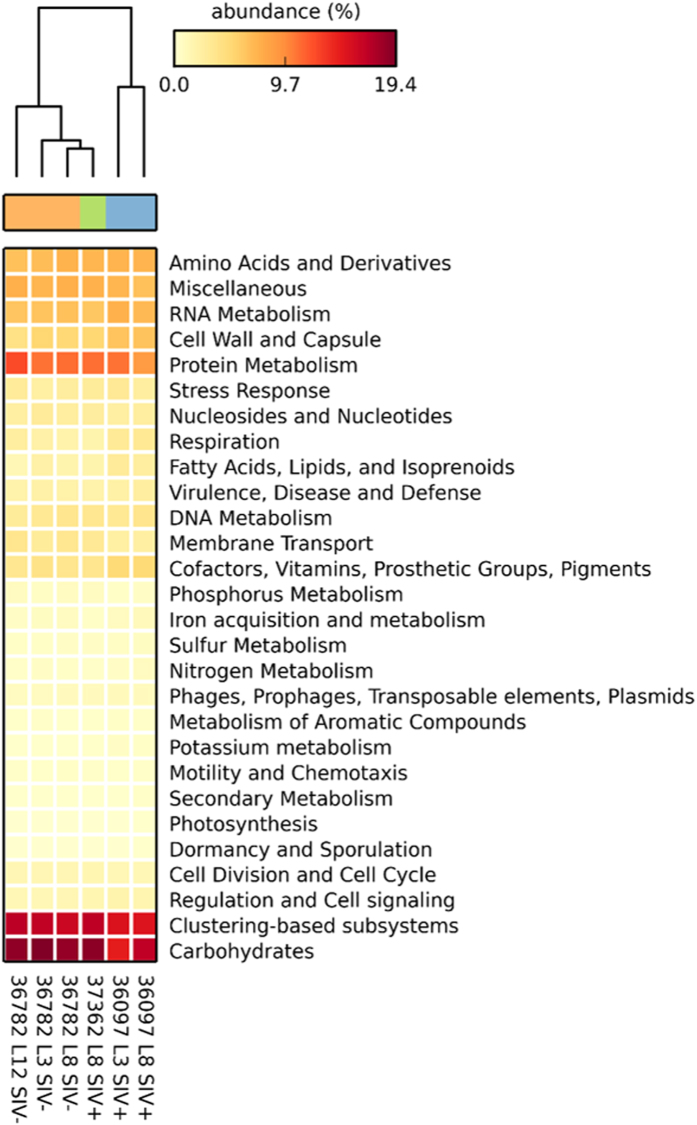
Hierarchical clustering and heatmap based on functional annotations from ileal metatranscriptomes. Functional annotations were determined by MG-RAST using the SEED Subsystems database with a maximum e-value of 1e-5, a minimum identify of 60%, and a minimum alignment length of 15 amino acids for translated protein sequences. STAMP was used to calculate relative abundances for the heatmap and perform hierarchical clustering using the unweighted pair group method with arithmetic mean (UPGMA) method.

The metatranscriptomes (excluding reads mapped to the rhesus macaque and *L. plantarum* genomes) were annotated with the MG-RAST server using the SEED Subsystems database and the top-level hierarchy was used to determine categories of transcripts present (Supplementary Table 2). For all metatranscriptomes, the highest number of transcripts were found for the carbohydrate metabolism, clustering-based subsystem (unknown function), and protein metabolism categories (Fig. 4). Hierarchical clustering analysis revealed that the transcripts grouped by animal and dominance of *Streptococcaceae* and *Pasteurellaceae* families but not by disease status (Fig. 4).

### Evidence of host-derived glycan utilization by gut-resident bacteria

**Figure 5:**
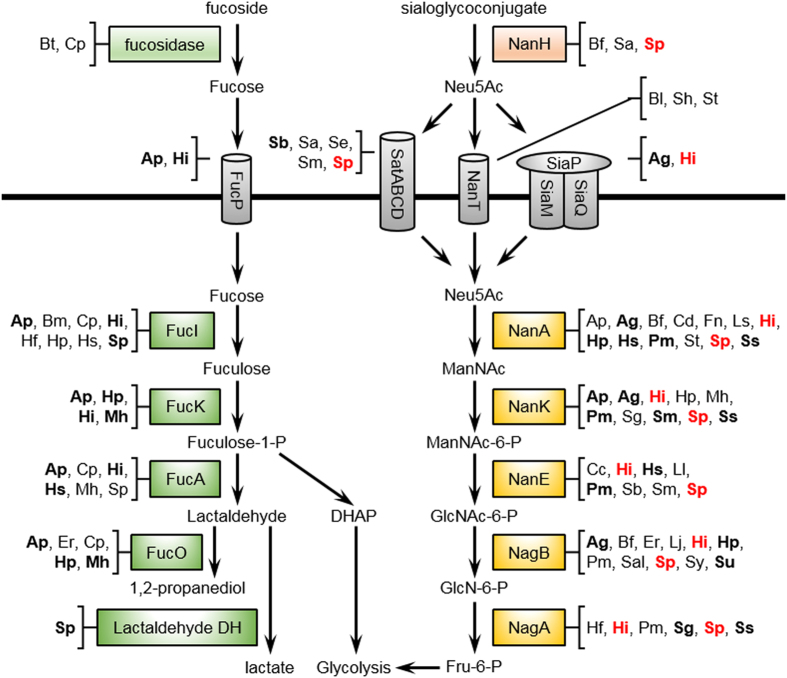
Host-derived glycan utilization pathways predicited by metatranscriptome analysis. Shown are the predicted general pathways for the utilization fucose and sialic acid (N-acetylneuraminate) by the rhesus macaque commensal microbiota. Species in bold indicate a transcript from that species was detected in at least two loops. Species in red indicate a complete sialic acid metabolism pathway was found. Abbreviations are as follows: Ap, *Actinobacillus pleuropneumoniae*; Ag, *Aggregatibacter actinomycetemcomitans*; Bf, *Bacteriodes fragilis*; Bt, *B. thetaiotaomicron*; Bl, *Brevibacterium* linens; Bm, *Brachyspira murdochii*; Cp, *Clostrium perfringens*; Cd, *Clostridium difficile*; Cc, *Cryptobacterium curtum*; Er, *Eubacterium rectale*; Fn, *Fusobacterium nucleatum*; Lj, *Lactobacillus johnsonii*; Ls, *L. salivarius*; Ll, *Lactococcus lactis*; Hi, *Haemophilus influenza*; Hp, *H. parasuis*; Hs, *H. somnus*; Hf, *Holdemania filiformis*; Mh, *Mannheimia haemolytica*; Pm, *Pasteurella multocida*; Sal, *Salmonella enterica*; St, *Staphylococcus aureus*; Sh, *S. haemolyticus*; Sb, *Streptobacillus moniliformis*; Sa, *Streptococcus agalactiae*; Se, *S. equi*; Sg, *S. gordonii*; Sm, *S. mitis*; Sp, *S. pneumonia*; Sy, *S. pyogenes*; Ss, *S. sanguinis*; Su, *S. suis.* Protein abbreviations are as follows: FucP, fucose permease; FucI, fucose isomerase; FucK, fuculokinase; FucA, fuculose phosphate aldolase; FucO, lactaldehyde reductase; NanH, sialidase; SatABCD, sialic acid ABC transporter; NanT, sialic acid transporter; SiaPQM, TRAP-type sialic acid transporter; NanA, N-acetylneuraminate lyase; NanK, N-acetylmannosamine kinase; NanE, N-acetylmannosamine-6-phosphate 2-epimerase; NagB, glucosamine-6-phosphate deaminase; NagA, N-acetylglucosamine-6-phosphate deacetylase.

Bacteria isolated from the intestine often possess the capacity to metabolize host-derived glycans such as sialic acid, fucose, and aminosugars^26^. Concordantly, here we detected transcripts originating from the small intestine microbiota in all three macaques showing that these bacteria are actively metabolizing these compounds *in vivo*. We were able to completely reconstruct the predicted pathway for sialic acid utilization from *Streptococcus pneumonia* and *Haemophilus influenza* (Fig. 5). This pathway includes extracellular sialidases, transport systems, and enzymes to shuttle sialic acid into glycolytic metabolism (Fig. 5). Transcripts for the consumption of sialic acid were also detected from a variety of other bacterial species belonging to different phyla (Fig. 5). Although mRNAs for the entire pathway were not detected for those bacteria, genomes of members of these species are annotated to contain all the genes necessary for the degradation of sialic acid. Similarly, partial pathways for the metabolism of fucose, another component of mucin, could be reconstructed based on detected transcripts from multiple bacterial species (Fig. 5). The bacteria from which these transcripts originated were in agreement with the proportions of the *Streptococcaceae* and *Pasteurellaceae* present.

### B vitamin biosynthesis by ileal microbiota

The rhesus macaque small intestine was enriched for bacterial transcripts coding for the synthesis of B vitamins. mRNAs coding for enzymes in the cobalamin (B_12_) biosynthetic pathway were detected for several species including cobalt-precorrin-6x reductase from *Veillonella parvula*, cobalt-precorrin-6A synthase from *Clostridium difficil*e, and cobalt-precorrin-2 C20-methyltransferase from *Streptococcus sanguinis* (Supplementary Table 2). Genomes from these species contain all the genes necessary for cobalamin biosynthesis which indicates that these bacteria might be important producers of cobalamin in their mammalian hosts. Transcripts for thiamine (B_1_), riboflavin (B_2_), pyridoxine (B_6_), biotin (B_7_), and folate (B_9_) biosynthetic enzymes originating primarily from *Pasteurellaceae* and *Streptococcaceae* were also identified (Supplementary Table 2).

## Discussion

Dietary and indigenous bacteria are under constant selective pressure in the different compartments of the digestive tract. The capacity of these bacteria to rapidly respond to changes in the intestinal environment is largely the result of rerouting gene expression to produce cell products used to tolerate host immune and epithelial cell-derived factors and the other microbes present. In this study, we found that shortly following SIV infection, the ileal lumen is not so altered compared to healthy animals as to necessitate transcriptome remodeling by *L. plantarum* WCFS1. Instead, *L. plantarum* adapts equally to the healthy and early SIV+ ileal environment by altering expression of carbohydrate metabolism, stress response, and cell-surface modification pathways. Remarkably, the indigenous microbiota were activated in similar pathways and were also seemingly unaffected by SIV infection in the presence of *L. plantarum.*

We examined the transcriptomes of *L. plantarum* WCFS1 in the ileum of healthy and SIV+ rhesus macaques to those measured for reference cultures consisting of exponential-phase *L. plantarum* cells in standard laboratory culture medium. These comparisons supported our conclusion that *L. plantarum* WCFS1 gene expression in the small intestine was highly similar between healthy and early SIV infected animals. Moreover, prior studies also used reference laboratory cultures to quantify relative changes in *L. plantarum* gene expression in the mammalian intestine. Notably here, we found that certain *L. plantarum* genes and pathways were induced in the ileum that were not recognized as *in vivo* inducible in the cecum and colon^11^,^12^,^13^,^15^. Such differences in transcript levels are supported by the finding that *L. plantarum* gene expression can vary depending on the intestinal site in which it is located^12^.

Prominent among the responses of *L. plantarum* specifically to the ileum was the induction of stress-response pathways. The majority of those up-regulated genes are responsible for coping with oxidative stress^22^,^27^. These genes have been characterized for indigenous bacterial colonists of the mammalian small intestine^28^,^29^,^30^. Notably, this response was unlikely to be due to the preparation and delivery of *L. plantarum* because strain WCFS1 is able to tolerate ambient oxygen levels better than other lactobacilli and care was taken to minimize exposure of the *L. plantarum* culture to air prior to injection. Taken together, this finding supports the premise that the ileum is at least a moderately oxidative environment to both dietary and native colonists.

The epithelium of the small intestine contains a mucus layer comprised of mucin glycoproteins that protects the epithelium and serves as a nutrient source or attachment site for resident bacteria^31^. To this regard, the *in vivo* expression of the mucin binding protein lp_1697 might indicate that this protein is used by *L. plantarum* WCFS1 to attach to mucus and maintain residency in the small intestine. Because *L. plantarum* lp_1697 binds to *N*-acetylglucosamine^24^, this amino sugar could be the specific constituent in mucin used for adherence. Other cell-surface associated genes were down-regulated in the ileum. For example, genes coding for capsular polysaccharide production by *L. plantarum* were repressed. These genes were induced by *L. plantarum* in the human colon^11^ but not the mouse cecum^13^. Deletion of the four capsular polysaccharide gene clusters (*cps1-4*) in *L. plantarum* previously led to increased NF-κB activation in cell cultures through TLR2 most likely because of a reduced shielding of lipoteichoic acid (LTA)^32^. Despite repression of most of the *cps* genes in rhesus macaques, the anti-inflammatory properties of *L. plantarum* were maintained and no activation of NF-κB was observed^20^. Therefore, it is possible that at even low expression levels, *L. plantarum* LTA is still shielded from TLR2 by CPS and only a complete abolition of the *cps* genes would result in NF-κB activation. Taken together, the variation in expression of *L. plantarum* genes coding for extracellular constituents strongly indicates that this organism modifies its cell surface *in vivo* in ways that alter its interactions with the host.

Transcript levels of *L. plantarum* WCFS1 genes required for mono- and di-saccharide utilization were lower in the small intestine than the reference cultures. Instead, the *L. plantarum* transcriptome was activated for the utilization of host-derived glycans. Genes for sialic acid metabolism and transport of *N*-acetylglucosamine/galactosamine (*pts19ADCB*) were induced in the macaque small intestine. That transporter was also induced by *L. plantarum* in the mouse cecum^13^,^14^,^15^, and the absence of this locus resulted in increased IL-10 production in human immune cells^33^. In agreement with the altered carbohydrate metabolism of *L. plantarum* in the small intestine was the induction of genes for the production of fumarate/malate (*fum*), formate (*pfl*), and ethanol (*adhE*) (Supplementary Table 1). Although *L. plantarum* homofermentative metabolism by the Embden-Meyerhof-Parnas pathway typically leads to the reduction of pyruvate to lactic acid, glucose limitation and non-preferred carbon sources can result in NAD(P)+ regeneration through the formation of these other end-products^34^,^35^.

In contrast to the limited expression of carbohydrate metabolism genes here, the opposite result was found for strains of this species in the large intestine of human and murine hosts^11^,^13^. Such differences could be due to variations in host environments encountered by *L. plantarum* or stimulated as a result of fasting the macaques prior to laparotomy and thereby restricting *L. plantarum* access to those sugars for growth. Also, the expression of some genes for carbohydrate metabolism might have been missed as a result of our transcriptome comparisons to *L. plantarum* MRS reference cultures. However, we expect this effect was minimal because the carbon-catabolite repression that occurs in glucose-replete medium such as MRS typically limits the extent to which *L. plantarum* expresses genes required for metabolism of other sugars^35^.

The metatranscriptomes showed that members of the *Streptococcaceae* and *Pasteurellaceae* families were the most metabolically active bacteria in the *L. plantarum*-treated macaque ileum. *Pasteurellaceae* and *Streptococcaceae* are both residents of the rhesus macaque small intestine^36^ and *Streptococcaceae* is normally found in the human small intestine^4^. These ileal microbial families rely on simple soluble glycans for growth as opposed to the predominant bacterial taxa in the large intestine that are better suited for degradation of complex polysaccharides^4^,^26^. Specifically, the ileal microbial families were likely consuming host-derived glycans as indicated by the complete and partial reconstruction of the sialic acid and fucose utilization pathways, respectively. That those transcripts in rhesus macaques primarily originated from either *Streptococcaceae* or *Pasteurellaceae* and concordant expression of the *N*-acetylneuraminate lyase by *L. plantarum* WCFS1 is strongly indicative of functional redundancy in the ileum for important metabolic pathways shared across indigenous species.

Metagenomic analyses indicate that the small intestine microbiota serve as suppliers of essential amino acids and vitamins^37^,^38^. We detected transcripts responsible for the biosynthesis of cobalamin, indicating that the microbiota in the ileum are producing cobalamin. Cobalamin is an essential vitamin for human health that is typically regarded to be acquired in foods^39^ and absorbed by the epithelia of the small intestine^40^. This vitamin is only produced by microorganisms and its production by the resident gastrointestinal bacteria of humans has been proposed but not empirically demonstrated^41^,^42^. The ileal microbiota may also serve as a source of the other B vitamins, although these vitamins are not exclusively produced by microorganisms and can be obtained from a variety of dietary sources.

The invasiveness of sampling the small intestine limits our understanding of the entirety of the human digestive tract^2^. By employing a non-human primate model, we circumvented this limitation and, even with the difficulties inherent to isolating RNA from the lumen of the small intestine, provide insight into the activity of the commensal microbiota in the ileum during the early stages of SIV infection. Only 2.5 days after the start of SIV infection, IL-1β production is increased by Paneth cells resulting in weakening of the epithelium^20^. Subsequent disease progression leads to further disruption of the intestinal epithelial barrier and dysfunctional mucosal immune responses resulting in microbial translocation and chronic inflammation^43^,^44^,^45^. Our results demonstrate that early on in SIV infection, commensal bacterial gene expression in the ileum does not respond to changes in the host’s enteropathy. Despite SIV-induced pressures for intestinal inflammation and impairment of the epithelial barrier, *L. plantarum* cell activity is identical to healthy animals while retaining its immunomodulatory properties and triggering reparation of the epithelium. Correspondingly, the indigenous rhesus macaque microbiota was also unaffected in the presence of the virus. Because all animals used in the study were treated with *L. plantarum*, we cannot determine how exactly the other bacteria respond to its presence. However, the effects of SIV infection were clearly minor as the ileal bacteria of both healthy and SIV+ animals were activated for the production of the same nutritive pathways. By understanding the cues for bacterial adaptation to the small intestine, we can elucidate the events leading to the perturbation of the mucosa during chronic stages of HIV infection while developing novel strategies for mitigating those effects through manipulation of the indigenous microbiome.

## Methods

### Bacterial strains and culture conditions

*L. plantarum* WCFS1 was maintained as a frozen glycerol stock at −80 °C. Bacteria were routinely cultured at 37 °C without agitation in MRS broth (BD, Franklin Lakes, NJ) or on MRS agar (1.5% wt/vol) plates.

### Animal experiments

Ligated ileal loops derived from a total of 8 male rhesus macaques (ages 3 – 6 years) in our previous study^20^ were used in this study. Four animals were inoculated intravenously with 1000 TCID_50_ of SIVmac251 for 2.5 d and the other 4 animals served as healthy negative controls. Animals were fasted for 36 h and subjected to ligated ileal loop surgery as previously described^20^,^44^. One ml of stationary phase *L. plantarum* containing 10^9^ colony-forming units (CFU) cultured in MRS broth was injected directly into the lumen of 3 ileal loops for each animal for a total of 24 loops. In order to retain any secreted metabolites or proteins made by *L. plantarum* in MRS, the cells were not washed prior to administration. Intestinal loops were collected 5 h after *L. plantarum* inoculation and the luminal contents were saved for RNA extraction.

### Ethics statement

This study was carried out in strict accordance with the recommendations of the Public Health Services (PHS) Policy on Humane Care and Use of Laboratory Animals. All animals were housed at the California National Primate Research Center. All animal procedures were performed according to a protocol approved by the Institutional Animal Care and Use Committee of the University of California, Davis. Appropriate sedatives, anesthetics and analgesics were used during handling and surgical manipulations to ensure minimal pain, suffering, and distress to animals. Furthermore, housing, feeding, and environmental enrichment were in accord with recommendations of the Weatherall report. Animals were euthanized in accordance with the American Veterinary Medical Association (AVMA) Guidelines for the Euthanasia of Animals (Section 2.3).

### RNA sequencing sample collection and RNA extraction

Luminal contents for ileal loops inoculated with 10^9^ CFU of *L. plantarum* WCFS1 were harvested after 5 hours of incubation. Also, luminal contents from one loop that was inoculated with only MRS broth (loop position 5 from animal 36097, see supplementary material from ref. 20) was also collected solely as a negative control to confirm the absence of *L. plantarum* WCFS1 or closely related strains in the ileum of the animals by RNA sequencing. Luminal contents were stored on ice, centrifuged at 1800 rpm for 5 min, and supernatants were snap frozen in liquid nitrogen. *L. plantarum* WCFS1 grown in MRS without agitation at 37 °C was harvested at OD_600_ of 1.0. The bacteria were centrifuged, the supernatant was removed, and the cell pellet was snap frozen in liquid nitrogen. All samples were stored at −80 °C until extraction. For total RNA extraction, two volumes of cold acidic phenol:chloroform:isoamyl alcohol [125:24:1] (Ambion, Carlsbad, CA, USA) were added to cryopreserved luminal content samples and thawed on ice. Next, 600 μl of the thawed luminal contents and phenol:chloroform:IAA mixture was transferred to a 2 ml screw-cap tube containing 500 buffer (200 mM NaCl, 20 mM EDTA), 210 μl 20% SDS, and 300 mg 0.1 mm zirconia/silica beads. Contents were mechanically lysed in a Fastprep-24 bead beater (MP Biomedicals, Santa Ana, CA, USA) by homogenizing twice at 6.5 m/s for 1 min with 1 min on ice between runs. The tubes were centrifuged at 7,600 *g* at 4 °C for 3 min and the upper aqueous phase was transferred to a new tube. An equal volume of cold acidic phenol:chloroform:IAA was added and the tubes were mixed by inversion and centrifuged as above. The upper aqueous phase was transferred to a new tube and the RNA was concentrated by isopropanol precipitation. RNA was quantified spectrophotometrically using a NanoDrop 2000c (Thermo Scientific, Waltham, MA, USA). DNase treatment was performed on 5 μg total RNA using the Turbo DNA-free kit (Ambion, Carlsbad, CA, USA). RNA quality was assessed using a Bioanalyzer RNA 6000 Nano Kit (Agilent Technologies, Santa Clara, CA, USA). A lack of contaminating DNA was confirmed by quantitative-reverse-transcriptase-PCR for *rpoB*^12^. RNA was extracted from all loops depicted in Fig. 1 however only RNA of sufficient quality (23S to 16S ratio greater than 1.5) was used for RNA-seq library construction.

### RNA-seq library construction and transcriptome analysis

Bacterial ribosomal RNA was removed from using the Ribo-Zero Magnetic Kit for bacteria (Epicentre, Madison, WI, USA) according to the manufacturer’s instructions. We did not attempt to remove host rRNA to minimize sample handling. mRNA was then purified using an RNeasy MinElute Cleanup Kit (Qiagen, Valencia, CA, USA) and the quality of the remaining mRNA was checked using a Bioanalyzer RNA 6000 Pico Kit and quantified by Qubit RNA HS Assay (Life Technologies, Carlsbad, CA, USA). RNA was reverse transcribed using SuperScript II enzyme (Life Technologies, Carlsbad, CA, USA) to produced 1^st^ strand cDNA. 2^nd^ strand cDNA was then synthesized using the NEBNext mRNA Second Strand Synthesis Module (New England Biolabs, Ipswich, MA, USA) and purified using a DNeasy MinElute Reaction Cleanup Kit (Qiagen, Valencia, CA, USA). cDNA was then sonicated using a Bioruptor NGS (Diagenode, Denville, NJ, USA) to produce 300 to 500 bp fragments. Fragmented cDNA was used to construct barcoded RNA-seq libraries using the NEXTflex ChIP-Seq Kit (Bioo Scientific, Austin, TX, USA) using gel-free size selection as per the manufacturer’s instructions. Size-selection was performed for approximately 300 bp inserts using Agencourt AMPure XP magnetic beads (Beckman Coulter, Indianapolis, IN, USA). Libraries were barcoded using NEXTflex ChIP-Seq barcodes. The library insert size distribution was confirmed using a Bioanalyzer High Sensitivity DNA Kit, quantified by Qubit HS DNA kit, and pooled in equal amounts. Libraries were then randomized and evenly distributed across 3 lanes of a HiSeq 2500 (Illumina, San Diego, CA, USA) for 50 bp single-read sequencing at the UC Davis DNA Technologies Core (http://dnatech.genomecenter.ucdavis.edu/).

Total RNA of suitable quality for RNA-seq was obtained from one loop each from animals 38299, 36171, and 37248 and three loops from animal 36782 (healthy) and 1 loop each from animals 35875, 36001, and 37362 and 2 loops from animal 36097 (SIV+ animals) (Fig. 1). We also extracted suitable RNA from one MRS-injected loop from animal 36097 (SIV+) (not shown). An average of 16,910,956 raw reads per RNA-seq library were obtained totaling over 12.6 Gbp (Supplementary Table 3). Raw reads were quality filtered and initially aligned against the rhesus macaque genome (NCBI RefSeq assembly accession GCF_000002255.3) using Bowtie2^46^ in the [–senstive] mode. Among those reads, 32.5 ± 24.8% aligned to the rhesus macaque genome and were discarded (Fig. 1 and Supplementary Table 3). The remaining reads were aligned to the *L. plantarum* WCFS1 chromosome and plasmids^47^ also using Bowtie2 in the [–sensitive] mode and among those reads, 47.5 ± 29.0% aligned to the WCFS1 genome. For the negative control loop 5 from animal 36097 inoculated with only MRS broth, only 0.02% of the reads aligned to the *L. plantarum* genome and these were distributed evenly across the genome (0.34 reads per locus) indicating very low levels of background *L. plantarum* in the animals (Supplementary Table 4). Reads which did not align to either the rhesus macaque or *L. plantarum* genomes were saved for metatranscriptome assembly and analysis (discussed below) (Fig. 1). Genome sequence alignments for *L. plantarum* cells grown in MRS were 88.9 ± 2.5%) and resulted in a genome coverage of 181.6 ± 15.6-fold (Supplementary Table 3).

*L. plantarum* aligned read counts (excluding those which aligned to either rRNA or tRNA sequences) were generated using HTSeq-count^48^. Differential gene expression based on infection status was determined with DESeq2^49^ in the R statistical environment by comparing counts from acutely infected animals to uninfected animals or bacteria grown in MRS culture medium. Differential expression was considered significant if the False-discovery-rate (FDR)-adjusted *p-*value was less than 0.05 and a 2-fold or greater change in expression was observed. Genes were grouped in Clusters of Orthologous Groups (COGs) categories for analysis^50^.

For metatranscriptome analysis, reads not originating from the host nor *L. plantarum* were first assembled into contigs using Trinity^51^ with a minimum contig length of 100 bp. Three libraries [animal 36171 loop 12 (healthy), animal 38299 loop 12 (healthy), and animal 36001 loop 8 (SIV +)] were not assembled into metatranscriptomes because they had less than 1 million reads not originating from the rhesus macaque or *L. plantarum* genomes. The remaining eight libraries were assembled into contigs (Supplementary Table 3). Two libraries [originating from animals 37248 loop 3 (healthy) and 35875 loop 12 (SIV+)] failed to assemble and were excluded from further analysis (Supplementary Table 3). The average number of contigs assembled for the libraries was 24,321 ± 15,559 and the number of contigs for each library positively correlates (R^2^ = 0.78) with the number of reads used for assembly. The same reads were then aligned back to the assembled contigs using Bowtie2 and contig abundances were estimated using RSEM^52^. The average median and mean contig lengths were 133 ± 3 and 189 ± 10 bp respectively for successfully assembled contigs. On average, 74.3 ± 3.2% of the reads were used to generate the final assemblies. The resulting contigs and abundances were annotated and analyzed using MG-RAST^53^. Hierarchical clustering and heatmaps were generated using STAMP^54^.

### Data accession numbers

*L. plantarum* RNA-seq data are available in the NCBI Sequence Read Archive (SRA) under BioProject accession no. PRJNA288882. Metatranscriptome data analyzed using MG-RAST can be accessed online (http://metagenomics.anl.gov/linkin.cgi?project=12839) under accessions 4622698.3, 4622699.3, 4622700.3, 4622701.3, 4623237.3, and 4623238.3 (see Supplementary Table 3 for MG-RAST names).

## Additional Information

**How to cite this article**: Golomb, B. L. *et al*. Gene expression of *Lactobacillus plantarum* and the commensal microbiota in the ileum of healthy and early SIV-infected rhesus macaques. *Sci. Rep.* **6**, 24723; doi: 10.1038/srep24723 (2016).

## Supplementary Material

Supplementary Figure 1

Supplementary Table 1

Supplementary Table 2

Supplementary Table 3

Supplementary Table 4
