# Supplementary material for: Gene expression of Lactobacillus plantarum and the commensal microbiota in the ileum of healthy and early SIV-infected rhesus macaques
Source: Sci Rep. 2016 Apr 22;6:24723. doi: 10.1038/srep24723 (PMC4840379; doi:10.1038/srep24723)
Supplement: Supplementary Figure 1 [file srep24723-s1.pdf]

Supplementary Information

Gene expression of *Lactobacillus plantarum* and the commensal microbiota in the ileum of healthy and early SIV-infected rhesus macaques

Benjamin L. Golomb[1], Lauren A. Hirao[2], Satya Dandekar[2], and Maria L. Marco[1#]

[1]Department of Food Science and Technology, University of California, Davis, CA, USA

[2]Department of Medical Microbiology and Immunology, University of California, Davis, CA, USA

[#]Corresponding author:

Maria L. Marco

One Shields Avenue

University of California, Davis

Davis, CA 95616

Phone: 530-754-4893

Email: mmarco@ucdavis.edu

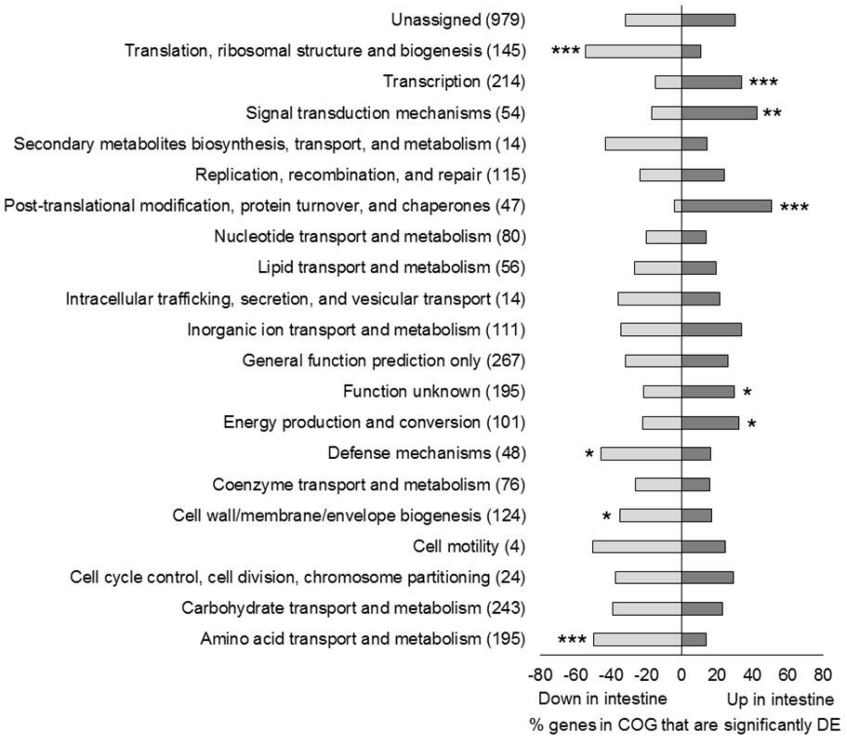

Supplementary Figure 1. COG categories of *L. plantarum* from SIV+ rhesus macaques. The percentage of *L. plantarum* genes in COG categories that were differentially expressed in the intestine of SIV+ rhesus macaques as compared to MRS is shown. The total number of genes in each COG are indicated in parentheses. Genes are considered differentially expressed (DE) if there was at least a 2-fold change in expression and an FDR-adjusted $P < 0.05$. COG categories that are significantly overrepresented according the χ^2 test compared to the total number of genes in the genome are indicated by an asterisk (*, $P < 0.05$; **, $P < 0.005$; ***, $P < 0.0005$).
